# A novel mouse model of heatstroke accounting for ambient temperature and relative humidity

**DOI:** 10.1186/s40560-021-00546-8

**Published:** 2021-04-16

**Authors:** Kazuyuki Miyamoto, Keisuke Suzuki, Hirokazu Ohtaki, Motoyasu Nakamura, Hiroki Yamaga, Masaharu Yagi, Kazuho Honda, Munetaka Hayashi, Kenji Dohi

**Affiliations:** 1 Department of Emergency, Critical Care and Disaster Medicine, Showa University School of Medicine, 1-5-8 Hatanodai, Shinagawa-ku, Tokyo, 142-8555 Japan; 2 Department of Anatomy, Showa University School of Medicine, 1-5-8 Hatanodai, Shinagawa-Ku, Tokyo, 142-8555 Japan

**Keywords:** Heatstroke, Animal model, Hot and humid circumstances, WetBulb globe temperature, Dehydration, Organ damage

## Abstract

**Background:**

Heatstroke is associated with exposure to high ambient temperature (AT) and relative humidity (RH), and an increased risk of organ damage or death. Previously proposed animal models of heatstroke disregard the impact of RH. Therefore, we aimed to establish and validate an animal model of heatstroke considering RH. To validate our model, we also examined the effect of hydration and investigated gene expression of cotransporter proteins in the intestinal membranes after heat exposure.

**Methods:**

Mildly dehydrated adult male C57/BL6J mice were subjected to three AT conditions (37 °C, 41 °C, or 43 °C) at RH > 99% and monitored with WetBulb globe temperature (WBGT) for 1 h. The survival rate, body weight, core body temperature, blood parameters, and histologically confirmed tissue damage were evaluated to establish a mouse heatstroke model. Then, the mice received no treatment, water, or oral rehydration solution (ORS) before and after heat exposure; subsequent organ damage was compared using our model. Thereafter, we investigated cotransporter protein gene expressions in the intestinal membranes of mice that received no treatment, water, or ORS.

**Results:**

The survival rates of mice exposed to ATs of 37 °C, 41 °C, and 43 °C were 100%, 83.3%, and 0%, respectively. From this result, we excluded AT43. Mice in the AT 41 °C group appeared to be more dehydrated than those in the AT 37 °C group. WBGT in the AT 41 °C group was > 44 °C; core body temperature in this group reached 41.3 ± 0.08 °C during heat exposure and decreased to 34.0 ± 0.18 °C, returning to baseline after 8 h which showed a biphasic thermal dysregulation response. The AT 41 °C group presented with greater hepatic, renal, and musculoskeletal damage than did the other groups. The impact of ORS on recovery was greater than that of water or no treatment. The administration of ORS with heat exposure increased cotransporter gene expression in the intestines and reduced heatstroke-related damage.

**Conclusions:**

We developed a novel mouse heatstroke model that considered AT and RH. We found that ORS administration improved inadequate circulation and reduced tissue injury by increasing cotransporter gene expression in the intestines.

## Background

Heatstroke is caused by exposure to high ambient temperature (AT) and can cause organ damage or death [1, 2]. Heatstroke incidences are increasing with the increase in global warming. The International Labour Organization has reported that AT is estimated to increase by 1.5–3.0 °C by 2100, which would result in increased frequency and severity of heatstroke worldwide; furthermore, heatstroke-associated economic losses are expected to exceed 2.4 billion USD by 2030 [3, 4].

Heat exposure is associated with damage to organs including the heart, lung, liver, kidney, gastrointestinal tract, muscle, and central nervous system. Moreover, it can induce systemic inflammatory response syndrome, which may lead to multiple organ dysfunction or death [1, 2, 5]. The human core body temperature (cT) is strictly maintained at 37.0 ± 1.0 °C. During exposure to high AT, the human body triggers heat divergence processes, including diaphoresis, increased respiratoryrate, and vasodilation, aiming to maintain the baseline cT. Among them, evaporation due to diaphoresis plays the most important role in the control of cT. However, this evaporation mechanism is impaired when the relative humidity (RH) is > 75% [6]. Therefore, people feel hotter in high ATs and RH than they would in dry conditions.

The WetBulb globe temperature (WBGT) is an environmental index that accounts for AT, RH, and the level of heat being radiated from the surroundings for assessing heatstroke risk [6]. The WBGT is used for decision-making and guideline development in sports, military training, etc. [7, 8].

Previously proposed animal models of heatstroke [9–13] disregarded the impact of RH, and created desert-like conditions, despite the general consensus that the WBGT can be used as a benchmark index. Therefore, we established a novel animal heatstroke model that considers AT and RH changes using conscious and unrestrained mice.

It is well known that heatstroke-induced severe fluid loss can cause splanchnic hypoperfusion and organ injury [14, 15]. Therefore, hydration is strongly recommended to prevent and treat heatstroke [16]. Drinking oral rehydration solution (ORS) is effective at treating fluid loss. The absorption of ORS is far superior to water as it is absorbed through the sodium/glucose cotransporter in the intestines [17]. Administration of ORS can relieve the severity of heatstroke symptoms in human patients [18]. Therefore, we examined the effect of hydration on heatstroke prevention and treatment to validate our model. Additionally, we investigated the gene expression of transporters in the intestinal membranes after heat exposure.

## Methods

Male C57/BL6J mice (aged 20–24 weeks) were used in this study. All animals were purchased from SLC Japan, Inc. (Shizuoka, Japan). The mice were allowed free access to food and water and were maintained on a 12-h light/dark cycle at room temperature (24 ± 2 °C) with constant humidity (40 ± 15%).

### Heat exposure protocol

**Fig. 1:**
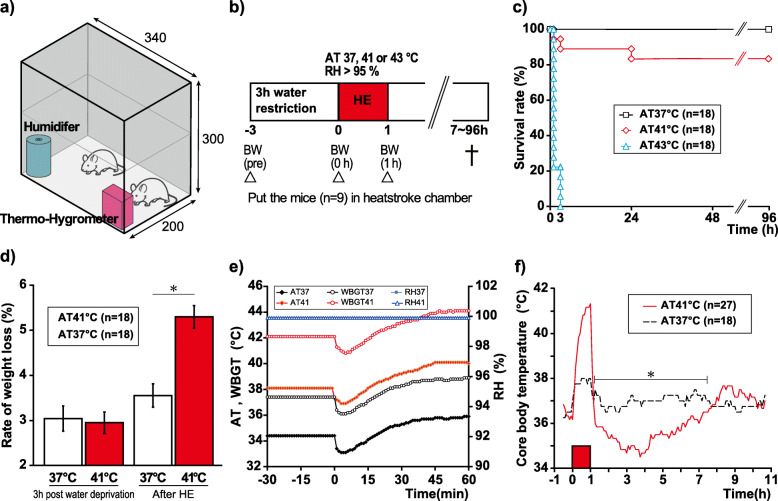
Experimental protocols and examination of conditions for our mice heat stroke model. **a** Heatstroke chamber: The heatstroke chamber was made using acrylic resin in a construction similar to a greenhouse. An ultrasonic humidifier was placed in the corner, and a thermo-hygrometer was used to monitor the environmental conditions. **b** Protocol for heatstroke: The mice (*n* = 9) were exposed to heat (ambient temperature 37 °C, 41 °C, or 43 °C) and relative humidity (> 99%) for 1 h and then returned to the chamber set to room temperature. They were sacrificed 7–96 h after heat exposure. **c** Survival rate (%) under three different ambient temperature (37 °C, 41 °C, or 43 °C) conditions observed during 96 h: All mice died within 3 h of exposure to the ambient temperature of 43 °C. The survival rate at the ambient temperature of 41 °C was 15/18 (83.3%). **d** Rate of body weight loss (%) at the ambient temperature of 37 °C and 41 °C: 3 h of water restriction induced approximately a 3% body weight loss at the ambient temperature of 37 °C and 41 °C. Body weight significantly decreased after 1 h of exposure to the ambient temperature of 41 °C, compared with that observed at the ambient temperature of 37 °C (*t* test, *p < 0.05). **e** WetBulb globe temperature (WBGT) and relative humidity under ambient temperature between 37 and 41 °C: WBGT always shows higher values than those of ambient temperature due to high humidity. RH was stabilized by more than 99.0% before and during the experiments. **f** Changes to core body temperature at ambient temperature of 37 °C and 41 °C: The core body temperature of the mice exposed to the ambient temperature of 41 °C increased markedly; subsequently, it decreased to 34.0 ± 0.18 °C (195 min after heat exposure). Then, the core body temperature gradually returned to physiological levels that showed biphasic thermal dysregulation response. There were significant differences in the core body temperatures measured during 1.0–7.4 h between the groups (*t* test, *p < 0.05).

A semi-enclosed heatstroke chamber (200 × 340 × 300 mm) made of acrylic was created by vertically stacking animal cages in a greenhouse-like construction. An ultrasonic humidifier (USB-68, Sanwa, Okayama, Japan) and a digital thermo-hygrometer (AD-5696, CA&D Company, Tokyo, Japan) were used for humidification and monitoring of the AT, RH, and WBGT (Fig. 1a). The heatstroke chamber was placed in an incubator (Bio-chamber, BCP-120F, TITEC, Aichi, Japan), which was pre-heated to the desired experimental temperature for ≥ 3 h. The humidifier was started 3 h before heat exposure to create a hot and humid environment. Meanwhile, the mice were given 3 h of water restriction and the mildly dehydrated mice were placed in the heatstroke chamber, exposed to heat for 60 min, and returned to the animal cage maintained at room temperature. The mice were euthanized 7–96 h after heat exposure. Nine mice were subjected to heat exposure in each experiment (Fig. 1b).

### Ambient temperature and heatstroke severity

We evaluated the extent of pathophysiological changes observed in the mice after exposure to different AT conditions. The mice were subjected to heat exposure at an AT of 37 °C (AT37 group), 41 °C (AT41 group), or 43 °C (AT43 group) and constant RH > 99% for 1 h. Survival rate for 96 h were observed and compared between the three groups (*n* = 18 per group). Body weight (BW) was measured thrice as an indicator of body fluid volume: before water restriction (pre), 3 h after water restriction and before heat exposure (BW 0h), and immediately after heat exposure (BW 1h; Fig. 1b). The weight loss rate (%) was calculated using the following formula (100 – BW [0 h or 1 h]/BW [pre] ×100). The animals that did not survive were excluded from this evaluation.

### Monitoring core body temperature

We used another set of mice to determine the cT during heat exposure. They were implanted with a small thermometer (Thermochrone type G, KN laboratories, Osaka, Japan) in their abdominal cavity as follows: (1) the mice were anesthetized using 4% sevoflurane in N_2_O/O_2_ (70/30%) inhalation, (2) an incision of approximately 1.0 cm was made on the abdominal midline under aseptic conditions, and (3) the thermometer was implanted between the abdominal aorta and intestinal membranes, and the incision was closed using sutures. The animals were maintained for 3 weeks for recovery and thereafter exposed to an AT of either 37 °C (*n* = 18) or 41 °C (*n* = 27). When the animals were euthanized, the thermometer was removed, and the cT records were transferred. The cT was recorded every 5 min during each experimental period after implantation.

### Evaluating organ injury

The animals underwent further examinations to evaluate organ injury 24 h after exposure to heat, including blood tests on samples collected from the right ventricle of the heart under sevoflurane anesthesia at 24 h after exposure to heat, and were perfused transcardially with 0.9% NaCl followed by 10%-neutralized formalin. Aliquots of blood were immediately examined for complete blood count (CBC) with VetScan HM5 (ABAXIS, Union City, CA, USA), while the remaining samples were centrifuged at 1500×*g* for 10 min to collect serum. Serum samples were examined for biochemical parameters: total protein (g/L), albumin (g/L), total bilirubin (umol/L), aspartate aminotransferase (IU/L), alanine aminotransferase (IU/L), alkaline phosphatase (IU/L), lactate dehydrogenase (IU/L), creatinine kinase (IU/L), blood urea nitrogen (mmol/L), creatinine (umol/L), sodium (mmol/L), potassium (mmol/L), chlorine (mmol/L), and glucose (mmol/L; HITACHI 7180, Tokyo, Japan).

Tissue samples from organs, such as the liver, kidneys, upper jejunum, and lungs, were extracted and prepared as paraffin-embedded sections of 5-μm thickness and evaluated for morphological changes using Hematoxylin-Eosin (HE) staining. One of the authors (KH; Pathologist) who was blinded to the experimental group assignment evaluated the specimens.

### Impact of oral rehydration solution on heatstroke

The mice were given ORS (OS-1®, Otsuka Pharmaceutical, Tokushima, Japan), tap water (water), or no treatment (NT) to validate our heatstroke model. Each experiment was performed with nine animals (3 mice/group) and repeated six times, with a total of 18 mice/group. We also prepared three other groups (5 mice/group: NT, water, and ORS) that were not exposed to heat to evaluate the hydration effect.

The mice were orally administered either ORS (30 mL/kg) or water (30 mL/kg) before and immediately after heat exposure. The NT group received no hydration during the experiment and was used as a control group. The animals were weighed and euthanized 7 h after heat exposure to allow enough time for fluid absorption [19]. The blood samples were collected; thereafter, tissue samples of six mice/group were perfused with 10% neutralized formalin for histological analysis. The upper jejuna were collected, snap-frozen in liquid nitrogen, and stored at − 80 °C for polymerase chain reaction (PCR) analysis.

### mRNA isolation and cDNA production

Isolation of total RNA and synthesis of cDNA were performed following the manufacturer’s instructions with minor modifications [20]. In brief, total RNA from the upper jejunum was isolated using TRIZOL Reagent (Invitrogen, Carlsbad City, CA, USA) and dissolved in RNase-free water. The purity and concentration of the extracted RNA were determined spectrophotometrically (NanoDrop, Wilmington, DE, USA). The cDNA was synthesized using 2 μg of total RNA with a High-Capacity RNA-to-cDNA kit (Applied Biosystems, Foster City, CA, USA).

### Polymerase chain reaction

We determined the gene expressions of sodium/glucose cotransporter 1 (SGLT1 encoded by *Slc5a1*), facilitated glucose transporter (GLUT2 encoded by *Slc2a2*), and intestinal fatty acid binding protein-2 (I-FABP encoded by *Fabp2*), which is known to contribute to intestinal injury markers. PCR analyses were performed using TaKaRa Ex Taq (TaKaRa, Shiga, Japan). All primers and gene information for *Slc5a1*, *Slc2a2*, *Fabp2*, and *Rplp1* (housekeeping gene) are presented in Table 1.

**Table 1:** Primers used in the present study

Name	Symbol	Forward (5′ to 3′)	Reverse (5′ to 3′)	Size (bp)
Rplp1	*Rplp1*	CTCGCTTGCATCTACTCCGC	AGAAAGGTTCGACGCTGACAC	109
Fabp2	*Fabp2*	TCCCTACAGTCTAGCAGACGG	CTCTCGGACAGCAATCAGCTC	118
Slc5a1	*Slc5a1*	ATGCGGCTGACATCTCAGTC	ACCAAGGCGTTCCATTCAAAG	247
Slc2a2	*Slc2a2*	ACTTGGAAGGATCAAAGCAATGT	CAGTCCTGAAATTAGCCCACAA	151

The reaction mixture was created with a suitable volume of the cDNA mixture, 0.25 μL of forward and reverse primers (50 nmol/mL), 2.0 μL of dNTP mixture (0.25 mM each), 0.1 μL of TaKaRa Ex Taq (5 units/μL), and 2.0 μL of 10×Ex Taq Buffer in a total volume of 20 μL. Thermal cycling parameters were set as follows: 95 °C for 1 min for initial denaturation followed by a cycling regimen of 40 cycles at 95 °C, 60 °C, and 72 °C for 45 s, 30 s, and 45 s, respectively. At the end of the final cycle, an additional 7-min extension step was included at 72 °C.

Quantitative PCR (qPCR) analyses were performed with SYBR Premix Ex Taq II reagent (TaKaRa), using the Applied Biosystems 7900HT Fast Real-Time PCR System (Applied Biosystems, Lincoln, CA, USA). The relative gene expression levels were calculated using the absolute quantification method against *Rplp1* (a housekeeping gene).

### Statistical analysis

Data were reported as the mean ± standard error of the mean. The Student’s *t* test was used for comparisons between two groups; one-way analysis of variance (ANOVA) and the Tukey–Kramer tests were used for multiple comparisons. *P* values < 0.05 were considered indicative of statistical significance.

## Results

### Survival rate and weight at different AT

Fourteen of the 18 mice in the AT43 group died during heat exposure; the remaining were unconscious and died within the following 3 h. Therefore, we excluded AT43 from the following assessments. All mice in the AT37 group survived for 96 h (18/18, 100%). In the AT41 group, the survival rate at 96 h was 15/18 (83.3%; Fig. 1c). As for weight, 3-h water deprivation induced around 3% BW loss, which indicated a mildly dehydrated state. Additionally, weight loss was significantly higher in the AT41 group than that in the AT37 group after 1 h of heat exposure (*t* test, *p* < 0.05; Fig. 1d).

### Alternation of AT and WBGT during heat exposure

The thermal conditions of the AT37 and AT41 groups subsequently increased and reached the peak at 35.9 ± 0.16 °C and 41.0 ± 0.11 °C, respectively, 60 min after heat exposure. WBGT always showed higher values than AT. WBGT finally increased to 38.9 ± 0.14 °C (AT 37) and 44.0 ± 0.15 °C (AT41) at 60 min, respectively. RH was stabilized to be more than 99.0% before and during the experiments (Fig. 1e).

### Impact of exposure to heat on core body temperature

The maximum cT of the AT37 group increased to 38.0 ± 0.09 °C during heat exposure and returned to its physiological level. Hypothermia after heat exposure was not seen in the AT37 group (Fig. 1f). However, the maximum cT of the AT41 group increased to 41.3 ± 0.08 °C during heat exposure and decreased to 34.0 ± 0.18 °C at 195 min thereafter. The AT41 group’s cT increased gradually and took approximately 8 h to return to physiological baseline levels after heat exposure, showing a biphasic thermal dysregulation response. There were significant between-group differences in the average cT recorded during 1.0–7.4 h (*t* tests, *p* < 0.05). Three animals in the AT41 group died and were excluded from further analysis.

### Impact of heat exposure on blood count and serum biochemical parameters

There was no between-group difference in the total red blood cell (RBC) or white blood cell (WBC) counts. The levels of hemoglobin (Hb) and hematocrit (Hct) significantly increased, while those of platelets (Plt) significantly decreased in the AT41 (*n* = 15) group compared with the AT37 (*n* = 18) group (*t* tests, *p* < 0.05).

Serum analysis revealed a significant increase in the levels of Na^+^ and Cl^-^ in the AT41 group. Moreover, serum biochemical parameters of the AT41 group revealed changes to the hepatic, renal, and musculoskeletal damage markers (Table 2).

**Table 2:** Blood parameters 24 h after exposure to ambient temperatures of 37 °C and 41 °C

n	37 °C	41 °C	37 °C
	18	15	41 °C(*)
**WBC × 10**^**6**^**(L)**	3235 ± 367	2425 ± 323	
**RBC× 10**^**12**^ **(L)**	9.1 ± 0.2	9.8 ± 0.2	
**Hb (g/L)**	132.4 ± 1.0	165.9 ± 2.2	*
**Hct (L)**	0.384 ± 0.003	0.476 ± 0.006	*
**Plt× 10**^**10**^ **(L)**	53.1 ± 2.2	35.3 ± 4.6	*
**TP (g/L)**	49 ± 0.6	59 ± 1.3	*
**Alb (g/L)**	33 ± 0.4	40.0 ± 0.6	*
**T-bil (umol/L)**	1.00 ± 0.17	0.86 ± 0.17	
**AST (IU/L)**	134 ± 20	907 ± 61	*
**ALT (IU/L)**	45 ± 4	282 ± 22	*
**ALP (IU/L)**	302 ± 10	746 ± 81	*
**LDH (IU/L)**	698 ± 165	4326 ± 223	*
**CK (IU/L)**	297 ± 36	5253 ± 215	*
**BUN (mmol/L)**	11.2 ± 0.44	17.5 ± 0.48	*
**Cre (umol/L)**	8.4 ± 0.27	40.1 ± 1.59	*
**Na (mmol/L)**	154.2 ± 0.7	160.4 ± 0.7	*
**K (mmol/L)**	4.8 ± 0.1	4.9 ± 0.3	
**Cl (mmol/L)**	117.5 ± 1.3	121.4 ± 1.1	*
**Glu (mmol/L)**	11.7 ± 0.66	15.1 ± 0.65	*

*p < 0.05

### Histopathological findings

**Fig. 2:**
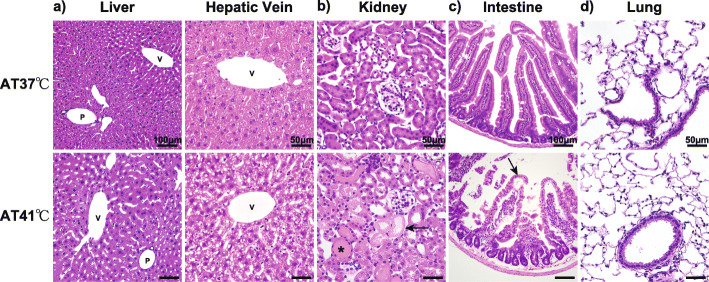
Histopathological findings of organ specimens collected after heat exposure. **a** Vacuolar hepatocytes (arrow) appeared around the hepatic central vein in the specimens of the animals exposed to the ambient temperature of 41 °C. P, portal vein; V, central vein. **b** Kidney specimens of the group exposed to the ambient temperature of 41 °C showed mild swelling and degeneration of tubular epithelial cells (arrow) and urinary casts (asterisk). **c** The intestinal structures of the group exposed to the ambient temperature of 41 °C were severely destroyed. The mucosal epithelial cells were eroded (arrow), and the intestinal villi showed interstitial edema (arrowhead). **d** No significant between-group differences were observed in the lung specimens of the group exposed to the ambient temperature of 37 °C and of that exposed to the ambient temperature of 41 °C

In contrast to liver specimens from the AT37 group, those from the AT41 group presented with vacuolar hepatocytes observed mainly around the hepatic central vein (Fig. 2a). The kidney specimens obtained from the AT41 group presented with mild swelling and degeneration of the tubular epithelial cells and the urinary cast (Fig. 2b). The intestinal structures of the AT41 group were severely damaged. The mucosal epithelial cells were eroded, and the intestinal villi showed interstitial edema (Fig. 2c). No between-group differences were observed in the lung specimens (Fig. 2d).

### Oral rehydration solution improved rehydration and reduced the tissue damage after heat exposure

**Fig. 3:**
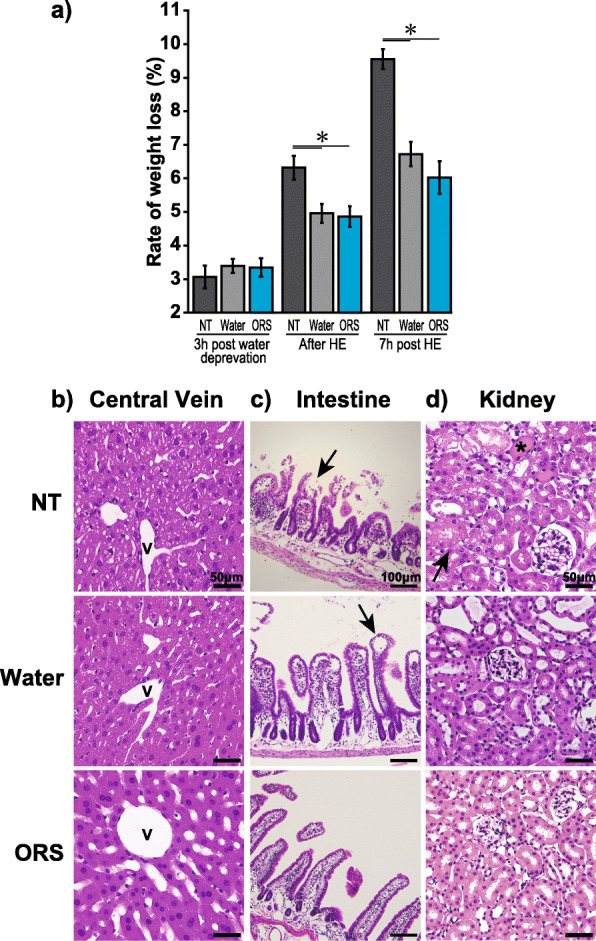
Effect of oral rehydration solution intake on body weight and histopathological findings of organ tissues. **a** Rate of weight loss (NT, water, ORS): The body weight of animals in the NT group was significantly reduced immediately and 6 h after exposure to heat (*p < 0.05). The use of water and oral solution had similar impact on the animals. **b** Hepatic vacuolation improved but remained present in the water group. Concurrently, there were very few formations in the oral rehydration solution group. **c** Intestinal tissue specimens from NT animals were marked with intestinal epithelial erosions (arrow) and swelling of the intestinal villi. Intestinal tissue specimens in the ORS showed only minor damage. **d** Renal tissue specimens in the NT group showed degeneration of the tubular epithelial cells (arrow) and urinary casts (asterisk). However, no damage was observed in the specimens acquired from the water and oral rehydration solution groups. NT, no treatment; ORS, oral rehydration solution; V, central vein

BW in the NT group significantly reduced immediately and 7 h after heat exposure compared to that in the water and ORS groups (Tukey–Kramer tests, *p* < 0.05; Fig. 3a). CBC and serum biochemical parameter levels in the NT group showed electrolyte abnormalities and hemoconcentration and significantly increased hepatic and renal damage markers than those observed in the other groups (Table 3). Compared to those of the water group, the levels of serum hepatic and renal damage markers in the ORS group were significantly lower (Tukey–Kramer tests, *p* < 0.05). The hepatic tissue specimens acquired from the NT group showed hepatic vacuolar degeneration mainly appeared around the hepatic vein. The hepatic vacuolation improved but remained present in the water group. However, very few formations were observed in the ORS group (Fig. 3b).

**Table 3:** Blood parameters of different intervention groups 6 h after heat exposure

n	NT	Water	ORS	NT	Water
	15	17	17	Water, ORS	ORS
**WBC ×10**^**6**^**(L)**	4128 ± 771	1776 ± 249	1505 ± 211	*	
**RBC×10**^**12**^ **(L)**	9.4 ± 0.4	8.6 ± 0.6	8.4 ± 0.5	*	
**Hb (g/L)**	165 ± 4.3	147 ± 2.5	142 ± 2.8	*	
**Hct (L)**	0.464 ± 0.016	0.423 ± 0.007	0.408 ± 0.008	*	
**Plt×10**^**10**^ **(L)**	36.9 ± 2.2	34 ± 3.2	37.2 ± 3.1		
**TP (g/L)**	55 ± 1.2	45 ± 0.8	43 ± 0.7	*	
**Alb (g/L)**	37 ± 0.6	31 ± 0.4	30 ± 0.5	*	
**T-bil (umol/L)**	1.40 ± 0.12	1.11 ± 0.10	1.0431 ± 0.10	*	
**AST (U/L)**	648 ± 36	614 ± 30	400 ± 17	*	*
**ALT (U/L)**	252 ± 16	262 ± 24	160 ± 13	*	*
**ALP (U/L)**	470 ± 11	361 ± 17	312 ± 18	*	
**LDH (U/L)**	3638 ± 157	3402 ± 129	1631 ± 113	*	*
**CK (U/L)**	5730 ± 357	5156 ± 216	5342 ± 261		
**BUN (mmol/L)**	33.8 ± 2.89	23.4 ± 2.03	14.7 ± 2.03	*	*
**Cre (umol/L)**	38.5 ± 11.49	13.4 ± 0.71	8.8 ± 0.53	*	*
**Na (mmol/L)**	162.1 ± 1.0	154.2 ± 0.7	154.0 ± 0.8	*	
**K (mmol/L)**	5.6 ± 0.2	4.5 ± 0.2	4.9 ± 0.1	*	
**Cl (mmol/L)**	115.6 ± 0.6	116.8 ± 0.7	116.8 ± 0.7		
**Glu (mmol/L)**	8.5 ± 0.57	7.7 ± 0.6	8.8 ± 0.49		

*NT* non-treatment, *water* tap water, *ORS* oral rehydration solution

*p < 0.05

The intestinal tissue specimens acquired from the NT group presented with intestinal epithelial erosions and swelling of the intestinal villi (Fig. 3c), while the intestinal tissue specimens of the water group showed edema of the lamina propria of the mucous membrane but normal intestinal villi. The intestinal tissues in the ORS group had hardly any damage. The renal tissue specimens from the NT group showed signs of degeneration of the tubular epithelial cells and urinary casts. However, no damage to these structures was observed in the water or in the ORS group (Fig. 3d). No changes to the pulmonary tissue were detected in any of the groups (data not shown).

### ORS administration with heat exposure significantly increased the gene expression of transporters in intestinal membranes

**Fig. 4:**
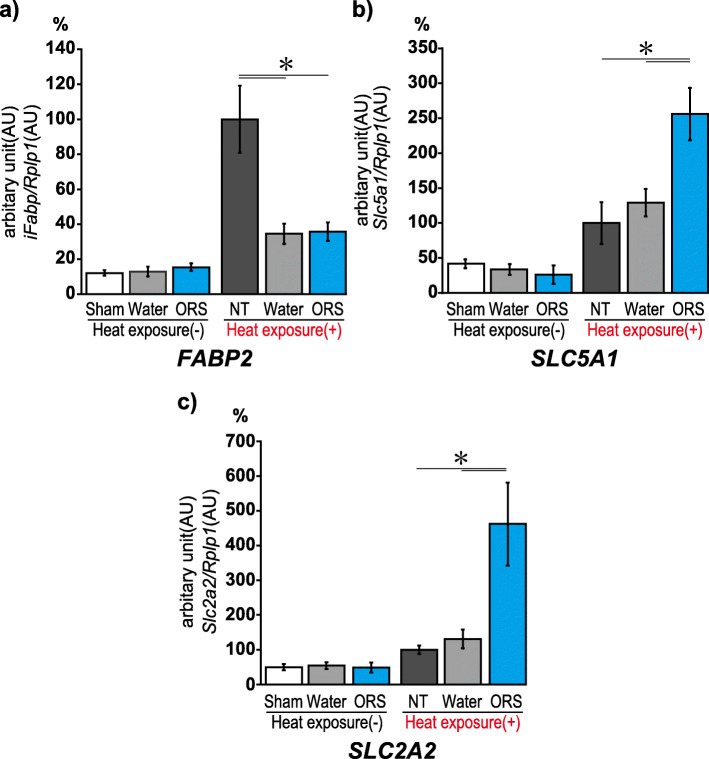
Expression of *Slc5a1*, *Slc2a2*, and *Fabp2* genes. **a** The level of *Fabp2* expression drastically increased in the non-beverage group 6 h after heat exposure. **b** The level of *Slc5a1* expression in the oral rehydration solution group was twice as high as that observed in the non-beverage and water groups. **c** The level of *Slc2a2* expression in oral rehydration solution group increased after heat exposure (*p < 0.05). Sham, normal mice; water (−), water intake without heat exposure; ORS (−), oral rehydration solution intake without heat exposure; NB (+), heat exposure without any beverage; water (+), water intake with heat exposure; ORS (+), oral rehydration solution intake with heat exposure

*Fabp2* expression levels increased in the NT group 7-h post-heat exposure but were suppressed in both the water and ORS groups without any inter-group differences (Tukey–Kramer tests, *p* < 0.05; Fig. 4a). *Slc5a1* expression levels increased significantly in all experimental groups after heat exposure. Concurrently, *Slc5a1* expression in the ORS group was significantly more than that in the NT and water groups (Fig. 4b). Finally, *Slc2a2* expression increased significantly more in the ORS group than in the NT and water groups (Fig. 4c). No significant changes were observed in the expression levels of the three genes without heat exposure.

## Discussion

Heat stroke mainly occurs in hot areas, although hot area varies from very low humidity deserts to hot and humid tropical regions as the world’s climate is highly diverse.

AT as well as RH plays an important role in the onset of heatstroke. For example, heatstroke is common even during the damp rainy days of early summer [21]. Therefore, we developed a mouse heatstroke model that mimicked temperate to subtropical regional weather conditions using WBGT as an indicator. In the monitoring of thermal conditions, WBGT always shows a higher value than the actual AT under hot and humid conditions. Heat-related deaths among outdoor workers and older adults have been reported at WBGTs above 33 °C [22]. In our model, the peak WBGT was 44.0 ± 0.15 °C during heat exposure. The thermal conditions in our study were more severe than those that induce heatstroke among humans.

In a previous study, Shen [9] reported a mouse heatstroke model with 42.4 °C AT and 50–55% RH for 1 h. However, in our study, many mice that were exposed to an AT of 43 °C and RH > 99% for 1 h died, and those that remained were in critical condition. The mortality rate was too high to consider it a viable experiment; therefore, we excluded the AT43 condition. It is known that the mechanisms of cT regulation in human and mice are different. Mice have fewer sweat glands than humans and are unable to regulate their body temperature through evaporation by perspiration [23]. Instead, they conduct heat and regulate body temperature through heat-vaporizing saliva and exhalation [24]. In our model, heat evaporation through vaporizing saliva and exhalation might not work effectively under the hot and humid condition that induced critical outcomes.

In our model, mice were subjected to a mildly dehydrated state by restricting water for 3 h prior to heat exposure. Dehydration is one of the important risk factors that aggravate heatstroke as it makes the subject prone to hypoperfusion [25]. In our model, BW decreased approximately 7–8% after 1 h of heat exposure indicating moderate to severe loss of body fluid volume. Therefore, a 3-h water restriction might be correlated with higher mortality in the AT43 group when compared with that in the other groups. Consequently, we reduced thermal conditions and performed heat exposure under 41 °C.

Several criteria for human heatstroke have been reported [2, 26]. A heatstroke in humans is defined as a cT > 40 °C and the presence of central nervous system (convulsive seizures), hepatic/renal, and coagulation dysfunction after exposure to high environmental temperatures. In a previously reported animal heatstroke model with conscious or unconscious subjects, the maximum cT during heat exposure was 40–43 °C [10–12]. Moreover, Leon [12] has reported that hypothermia developed after heat exposure is a biphasic thermoregulatory response and the depth and duration of hypothermia are correlated with the severity of heatstroke. This biphasic thermoregulatory response to heatstroke is also observed in humans [27]. Therefore, excessive cooling of heatstroke patients is not recommended as it sometimes induces hypothermia [28]. In our study, the average cT during heat exposure of the AT41 group reached a maximum of 41.3 °C and then decreased to a minimum of 34.0 °C. Therefore, a biphasic thermoregulatory response was observed and the maximum cT achieved was comparable to that reported in previous literature. Contrastingly, such a response was not seen in the AT37 group. Although a biphasic thermoregulatory response is theorized to occur due to hypothalamic impairment [29, 30], we did not determine the cause of thermal dysregulation in mice in this experiment.

Heat stress and inadequate circulation during heat exposure induces tissue damage, including hepatic, renal, and intestinal injuries in humans and animals [2, 31, 32]. Our results also showed an increase in tissue damage markers in the AT41 group, suggesting the occurrence of rhabdomyolysis and hepatic and renal damage. Subsequent histological findings of the hepatic and renal tissues extracted from the AT41 group also showed hepatic and renal damage after heat exposure. Particularly, vacuolar hepatocytes were present in abundance around the hepatic central vein and farthest from hepatic circulation. This supports the hypothesis that heat exposure may reduce blood circulation, resulting in tissue damage. The intestinal specimens from the AT41 group showed mucosal epithelial cell erosion and interstitial edema in the intestinal villi. Hall [15] has reported that splanchnic hypoperfusion may result in ischemia to the gastrointestinal organs, followed by a reperfusion injury during sudden splanchnic vasodilatation that precedes the onset of hemodynamic collapse and hyperthermia. Splanchnic hypoperfusion might correlate with the intestinal injury observed in our model.

Further, we examined the validity of our mouse heatstroke model by comparing different types of hydration (water/ORS). In our results, hydration improved hemoconcentration with no variation with the intervention type (water/ORS). However, the serum marker levels of hepatic and renal damage were significantly better in the ORS group than in the water group, suggesting that ORS might be more effective than water at suppressing heatstroke damage. Moreover, histopathological observations in the ORS group only showed minor tissue injury. A possible explanation is that ORS contains glucose and electrolytes, which improve absorption from the digestive tract through sodium/glucose cotransporters and improve tissue circulation [33, 34]. These results indicate that our model resembled the pathophysiology of a heatstroke experienced by a human.

Furthermore, we focused on cotransporter gene expression in the intestinal membranes to explore the effect of ORS after heatstroke. SGLT1 and GLUT2 are expressed in the apical and basolateral mucosal epithelial membranes of the small intestine. They co-transport glucose from the intestinal lumen into the capillaries in a process driven by the Na^+^ gradient created by Na^+^/K^+^ ATPase [35, 36]. Moreover, we investigated the gene expression of I-FABP as an intestinal ischemia marker after heatstroke. Plasma and urinary levels of I-FABP are reported to increase after intestinal ischemia [37]. Additionally, plasma I-FABP levels are increased in heatstroke patients [38]. In the present study, expression levels of all three genes were not increased exclusively by hydration; heat-exposed mice tended to express these genes more than mice that were not exposed to heat. *Fabp2* expression was upregulated in the NT group, suggesting an increase in the extent of intestinal ischemia post-heatstroke. Meanwhile, the expression levels of both SGLT1 and GLUT2 were significantly increased in the ORS group, suggesting that hydration with ORS increases the water and electrolyte absorption rates and may lead to improvement in hemodynamics and reversal of tissue damage. Further research is needed to explore the pathophysiology of heatstroke using this model.

Our study has some limitations. Firstly, mice have fewer sweat glands than humans and are unable to regulate their cT temperature through evaporation by perspiration. There are some reports in human heatstroke with 43 °C that have recovered completely [39]. The regulation of cT is different in humans and mice. Secondly, we usually give cold intravenous fluid and sometimes use continuous renal replacement therapy to control cT and remove myoglobin in clinical setting. The speed of reduction of cT is much faster in human heatstroke without having hypothermia. Lastly, we did not consider consciousness disturbance and coagulation abnormality in our model. Next, we will explore central nervous system injury due to heatstroke in another experiment using our model.

## Conclusion

In addition to AT, RH plays an important role in the onset of heatstroke. We developed a novel mouse heatstroke model which considered AT and RH used WBGT as an indicator. We found that ORS administration with heat exposure increased transporter gene expression (SGLT1 and GLUT2) in the intestinal membranes and reduced heatstroke-related damage. Adequate hydration with ORS before and after heat exposure may improve the symptoms of heatstroke patients.

## Data Availability

All data generated or analyzed during this study are included in this published article and its supplementary information files.

## Abbreviations

ANOVA: Analysis of variance
AT: Ambient temperature
BW: Body weight
CBC: Complete blood count
cT: Core body temperature
Hb: Hemoglobin
Hct: Hematocrit
HE: Hematoxylin-Eosin
NT: No treatment
ORS: Oral rehydration solution
PCR: Polymerase chain reaction
Plt: Platelets
qPCR: Quantitative polymerase chain reaction
RBC: Red blood cell
RH: Relative humidity
WBC: White blood cell
WBGT: WetBulb globe temperature
